# Impact of the COVID-19 virus outbreak on movement and play behaviours of Canadian children and youth: a national survey

**DOI:** 10.1186/s12966-020-00987-8

**Published:** 2020-07-06

**Authors:** Sarah A. Moore, Guy Faulkner, Ryan E. Rhodes, Mariana Brussoni, Tala Chulak-Bozzer, Leah J. Ferguson, Raktim Mitra, Norm O’Reilly, John C. Spence, Leigh M. Vanderloo, Mark S. Tremblay

**Affiliations:** 1 Department of Therapeutic Recreation, Faculty of Child, Family, and Community Studies, Douglas College, 1250 Pinetree Way, Coquitlam, BC V3B 7X3 Canada; 2 School of Health and Human Performance, Dalhousie University, PO Box 15000, Halifax, NS B3H 4R2 Canada; 3 School of Kinesiology, University of British Columbia, 6081 University Blvd, Vancouver, BC V6T 1Z1 Canada; 4 Behavioural Medicine Laboratory, School of Exercise Science, Physical and Health Education, University of Victoria, PO Box 1700 STN CSC, Victoria, BC V8W 2Y2 Canada; 5 Department of Pediatrics, University of British Columbia, 4480 Oak Street, Vancouver, BC V6H 0B3 Canada; 6 School of Population and Public Health, University of British Columbia, 2206 East Mall, Vancouver, BC V6T 1Z3 Canada; 7 ParticipACTION, 77 Bloor Street West, Suite 1205, Toronto, ON M5S 1M2 Canada; 8 College of Kinesiology, University of Saskatchewan, 87 Campus Drive, Saskatoon, SK S7N 5B2 Canada; 9 School of Urban and Regional Planning, Ryerson University, 105 Bond Street, 4th Floor, Toronto, ON M5B 2K3 Canada; 10 Gordon S. Lang School of Business and Economics, University of Guelph, 50 Stone Road East, Guelph, ON N1G 2W1 Canada; 11 Faculty of Kinesiology, Sport, and Recreation, University of Alberta, 1-153 Van Vliet Complex, Edmonton, AB T6G 2H9 Canada; 12 Child Health Evaluative Sciences, The Hospital for Sick Children, 555 University Avenue, Toronto, ON M5G 1X8 Canada; 13 Children’s Hospital of Eastern Ontario Research Institute, 401 Smyth Road, Ottawa, ON K1H 8L1 Canada; 14 Department of Pediatrics, University of Ottawa, 401 Smyth Road, Ottawa, ON K1H 8L1 Canada

## Abstract

**Background:**

Healthy childhood development is fostered through sufficient physical activity (PA; including time outdoors), limiting sedentary behaviours (SB), and adequate sleep; collectively known as movement behaviours. Though the COVID-19 virus outbreak has changed the daily lives of children and youth, it is unknown to what extent related restrictions may compromise the ability to play and meet movement behaviour recommendations. This secondary data analysis examined the immediate impacts of COVID-19 restrictions on movement and play behaviours in children and youth.

**Methods:**

A national sample of Canadian parents (*n* = 1472) of children (5–11 years) or youth (12–17 years) (54% girls) completed an online survey that assessed immediate changes in child movement and play behaviours during the COVID-19 outbreak. Behaviours included PA and play, SB, and sleep. Family demographics and parental factors that may influence movement behaviours were assessed. Correlations between behaviours and demographic and parental factors were determined. For open-ended questions, word frequency distributions were reported.

**Results:**

Only 4.8% (2.8% girls, 6.5% boys) of children and 0.6% (0.8% girls, 0.5% boys) of youth were meeting combined movement behaviour guidelines during COVID-19 restrictions. Children and youth had lower PA levels, less outside time, higher SB (including leisure screen time), and more sleep during the outbreak. Parental encouragement and support, parental engagement in PA, and family dog ownership were positively associated with healthy movement behaviours. Although families spent less time in PA and more time in SB, several parents reported adopting new hobbies or accessing new resources.

**Conclusions:**

This study provides evidence of immediate collateral consequences of the COVID-19 outbreak, demonstrating an adverse impact on the movement and play behaviours of Canadian children and youth. These findings can guide efforts to preserve and promote child health during the COVID-19 outbreak and crisis recovery period, and to inform strategies to mitigate potential harm during future pandemics.

## Introduction

Healthy movement behaviours contribute to the physical and mental health of children and youth [1] including a more robust immune system [2]. The evidence of movement behaviours for healthy growth and development is sufficiently compelling that many countries, including Canada [3], and the World Health Organization (WHO) [4] have released 24-h integrated movement behaviour guidelines for children and youth. These guidelines recommend age-specific physical activity, sedentary behaviour, and sleep thresholds for school-aged children and youth [3]. Children and youth who meet movement recommendations have better cardiometabolic, musculoskeletal, cognitive, and mental health, and immune function compared with their less active peers [1–4]. Despite the evidence indicating the benefits of increased physical activity, reduced sedentary behaviours, and adequate sleep, the prevalence of Canadian children and youth meeting the 24-h movement recommendations was recently reported to be only 12.7% [5]. Notwithstanding the notion that indoor physical activity can contribute to overall levels, the additional benefits of playing outside [6] and in nature are clear [7]. Spending time outside and in nature provides a critical venue for healthy movement behaviours, permitting children and youth to accumulate more daily physical activity, less sedentary behaviour, and sleep better [6].

On March 11, 2020, the WHO characterized the COVID-19 virus outbreak as a global pandemic [8]. COVID-19 is caused by a coronavirus which can result in acute respiratory distress in humans and is transmitted through respiratory droplets and contact routes [9]. Current estimates suggest a 1–2% case fatality rate, which varies by age and health of the patient [10]. Though healthy children and youth are less vulnerable to COVID-19 [11], there remains genuine concern about transmission of the virus, especially the spread to older people and those with underlying medical conditions. Consequently, the COVID-19 virus outbreak has led to significant changes in daily life for children, youth, and their families, with specific recommendations and restrictions varying within and between countries. Like many countries, Canada imposed restrictions requiring physical distancing (two metres), and limited community and social gatherings and interactions, sport, and playground and park use [12]. Most children and youth are no longer attending school, with classroom lessons replaced by home-schooling and online learning activities. During the initial response to the COVID-19 outbreak and recommendations for physical distancing, behaviour restrictions, and overall instructions to ‘stay home’, families are seeking guidance and solutions to preserve healthy routines, including healthy movement behaviours and opportunities to spend time outdoors [13, 14].

Although differences exist in the number of COVID-19 cases across geographic jurisdictions [15], preliminary data demonstrate that Canada is moving towards ‘flattening the curve’. [16] Federal and provincial politicians and Medical Officers of Health have continued to encourage Canadian families to get active and outdoors [17]. Yet, how the ‘stay home’ versus ‘get active and outdoors’ paradox is interpreted is unknown. Like the number of cases, COVID-19 restrictions and recommendations vary between jurisdictions [15]. It is timely to assess whether COVID-19 and related behavioural requirements have impacted the movement and play behaviours of Canadian children and youth, and to identify strategies that families are taking to stay healthy during this pandemic. Accordingly, the purpose of this secondary data analysis was to provide a rapid and large-scale assessment of the immediate changes in physical activity, play, sedentary behaviours, leisure screen time, and sleep in children and youth across Canada during the initial period of the COVID-19 crisis. We anticipate these finding will inform efforts to preserve and promote child health behaviours and establish priorities for post-COVID-19 public health initiatives.

## Methods

### Study design and population

ParticipACTION is a Canadian non-profit organization promoting physical activity. It recently conducted a survey (Additional File 1) assessing changes in children and youth movement behaviours during the COVID-19 virus outbreak to inform the upcoming release of its biennial Report Card of Physical Activity for Children and Youth. Accordingly, a cross-sectional sample of 1503 parents of Canadian children and youth aged 5–17 years was recruited via a third-party market research company. Maru/Matchbox has a consumer online database of > 120,000 Canadian panellists. Manu/Matchbox panel participants are recruited through a variety of online and offline methods and receive small cash incentives ($0.50–$3.00 CDN) and prize opportunities after completing surveys. The panel is comparable with the Canadian census in terms of age, gender, region, income, employment, and language spoken [18]. This sampling strategy is routinely employed by national organizations given the ability to rapidly recruit large, representative Canadian samples [19]. Maru/Matchbox panellists consent to participate in survey-based research when they sign-up for the panel. Subsequently, participants passively consented to participate when they agreed to complete the survey. This secondary data analysis was approved by the University of British Columbia’s Research Ethics Board (#H20–01371). In reporting this secondary data analysis the authors were attentive to the STROBE Statement (STROBE checklist; Additional File 2).

### Measures

#### Survey development

Health behaviours for children and youth were defined according to the Canadian 24-Hour Movement Guidelines for Children and Youth [1, 3]. Key content areas were developed by applying the socioecological model; the survey included parental and child demographics, current movement behaviours, change in child’s movement and play behaviours, and parental support for their child’s movement and play behaviours (Additional File 1). Selected items were used in this secondary analysis (Table 1). Test-retest (one week) reliability was assessed (similar to other ParticipACTION surveys [19]) via bivariate Pearson correlation (*n* = 100 participants). Strong reliability across 12 of the 13 items was demonstrated (correlation range = 0.79–0.99, magnitude of strength = .6 < | *r* | < .9, *p* < 0.001). A non-statistically significant linear relationship (correlation = 0.76, 0.53, *p* = 0.06) was reported for ‘inside physical activity’.

**Table 1:** Selected items used in the current analysis from the children and youth movement and play behaviours during COVID-19 virus outbreak survey (copy of full survey in Additional File 1)

Survey Details	
A. **Screening questions**	
***Question***	***Response option***
Has anyone in your household been diagnosed with COVID-19?	- Yes [thank you, terminate] - No [continue]
Is your household under a self-isolation or quarantine order?	- Yes [thank you, terminate] - No [continue]
B. **Demographic characteristics**	
**Parent:** Age and gender Geographic region Education; Marital status; Ethnicity Postal code Household make-up (number of adults/children)	[dropdown, age rollup] [dropdown, provinces rollup] [dropdown, specify] [dropdown, number rollup]
**Child:** Age and gender Disability or chronic condition Type of residence (e.g., house, apartment) Dog ownership	[dropdown, age rollup] [dropdown, specify] [dropdown, specify] [dropdown, yes/no]
C. **Current movement behaviours**	
In the last week, how many total hours and minutes per day did your child watch TV, use the computer, use social media and play inactive video games?	- Weekdays (hrs, mins) - Weekend days (hrs, mins)
In the last week, on how many days did your child engage in moderate-to-vigorous physical activity for a total of at least 60 min per day?	[dropdown, 0–7 days]
In the last week, how many hours did your child usually spend sleeping in a 24-h period (including naps but excluding time spent resting)?	[dropdown, 0–24 h]
D. **Change in movement and play behaviours**	
**Compared to before the COVID-19 outbreak and related restrictions:** - My child walks or bikes in the neighbourhood? - My child is doing physical activities or sport outside? - My child is doing physical activities or sport inside? - My child is doing household chores (e.g. cleaning, yard work)? - My child plays outside? - My child plays inside? - My child watches TV, movies, uses the computer for leisure? - My child uses social media? - My child does other sedentary leisure activities not in front of screens? - My child sleeps? - Our family time spent in physical activity is? - Our family time spent in sedentary behaviours is?	- A lot less - A little less - Same - A little more - A lot more
- My child’s sleep quality is? - The balance of my child’s overall healthy movement behaviours are? - My child’s overall time spent outside is?	- A lot worse - A little worse - Same - A little better - A lot better
**Compared to before the COVID-19 outbreak and related restrictions:** - Is there an inside leisure activity or hobby that your child is doing a lot more? - Is there an outside leisure activity or hobby that your child is doing a lot more - Has your family begun any new or novel activities not previously practiced? - Has your family used any online resources/apps to support healthy movement? - Has there been a decrease in your child’s health (e.g., existing condition worsened or new condition developed)?	- Yes [specify] - No
Do you have any advice for families trying to achieve a healthy balance of movement behaviours (physical activity, screen time, sleep) of their children during the COVID-19 outbreak and related restrictions?	- Yes [specify] - No

#### Survey content

Respondents provided parental and child demographic information and assessments of their child’s movement behaviours in the last week. They were subsequently asked to compare their child’s behaviours before and during the COVID-19 outbreak. Similarly, based on previous surveys, respondents were also asked to compare their support of their child’s movement behaviours before and during the COVID-19 outbreak. Responses were reported using a 5-point Likert type scale, ranging from ‘a lot less’ (score = 1) to ‘no change’ (score 3) to ‘a lot more’ (score = 5).

### Data collection

Approximately 1 month after the WHO declared COVID-19 a global pandemic [8], eligible participants were invited to complete an online survey (French or English). During recruitment, the sample was stratified by gender and age of the child (families with a child aged 5–11 years and families with a youth aged 12–17 years). Potential participants were screened out if they or their child had been diagnosed with COVID-19 or if the family was in self-isolation or quarantine due to COVID-19. Potential respondents were sent an email link to the survey, which required approximately 15 min to complete. Parents with more than one child were asked to think of the child whose given name came first alphabetically and to use that child as the referent for the survey (i.e., the index child). After data collection was complete, a cleaned dataset (.csv file) was received by ParticipACTION from Maru/Matchbox. Further data cleaning and verification was completed by the investigators (RER, SAM, GF, MST).

### Statistical analyses

#### Quantitative analyses, close-ended questions

Data were analysed in SPSS 20 (SPSS Inc., Chicago, IL, USA). Overall, age group, and gender specific means (standard deviations) for all variables were calculated. Factorial analyses of variance (by age-group and gender) were used to test for differences between continuous variables and chi-square tests were used to assess differences between categorical variables. Statistical significance was set at *p* < 0.01. The proportion of children meeting the Canadian 24-Hour Movement Behaviour Guidelines [3] was determined. Associations between movement and play behaviours and demographic and parental factors were assessed by Pearson and point-biserial correlation. Means (standard errors) were plotted for selected variables. Analyses were completed and checked by the analysis team (RER, SAM, GF, MST).

#### Qualitative analyses, open-ended questions

Two independent assessors (LJF, LMV) reviewed responses of the open-ended survey items. Given the brevity of responses, assessors adopted a word frequency distribution approach to review the data and elicit commonalities in responses across each of the questions [20]. Approximately 19.0% of responses were in French and translated to English by one of the coders (LMV). To establish reliability and consistency across coders, the first 100 responses of the ‘inside activities or hobbies’ question was independently coded by each assessor and then compared. A total agreement of 98.3% was achieved; discrepancies were due to differences in code labels (e.g., ‘making videos’ vs. ‘video production’) rather than a misattribution in classification by coders.

## Results

### Parent and child characteristics

Descriptive statistics for parent and child characteristics are provided in Table 2. A total of 1503 parents completed the survey. Respondents with missing or implausible data were excluded from these analyses (*n* = 31; e.g., parental age 15 years and child age 10 years). The final sample included 1472 participants. Geographic, ethnic, and age distribution of the sample was reflective of Canadian demographics. Respondents were primarily female (54.0%), married or cohabiting (84.2%), college or university graduates (88.7%), and working fulltime (70.1%). Type of home residence was predominantly a detached house (72.1%), and approximately one-third (35.4%) reported having a dog. Children (mean 8.1 years, range 5–11 years) and youth (mean 14.9 years, range 12–17 years) were primarily typically developing (91.4%). While most children and youth (71.1%) were meeting sleep recommendations, most were not meeting physical activity or screen time guidelines (18.2 and 11.3%, respectively). Only 2.6% of children and youth were meeting the combined 24-h movement behaviour (physical activity, sedentary behaviour, sleep) recommendations.

**Table 2:** Description of parent, child, and youth characteristics (*N* = 1472)

***Parent demographic profile***	
Age, M (SD)	45.12 (7.55)
Gender female, *n* (%)	689 (47.00)
Ethnicity, *n* (%)	
European	1166 (79.21)
Asian	194 (13.18)
Indigenous	61 (4.14)
Other	51 (3.46)
Marital status, *n* (%)	
Married or common-law	1239 (84.17)
Divorced or separated	153 (10.39)
Single	70 (4.76)
Widowed	10 (0.68)
Education, *n* (%)	
High school or less	166 (11.28)
College/Technical	558 (37.91)
University	508 (34.51)
Advanced degree	240 (16.30)
Annual household income, *n* (%)	
≤ $50,000	219 (14.88)
$51,000 to $99,000	499 (33.90)
$100,000+	586 (39.81)
Undisclosed	168 (11.41)
Employment status, *n* (%)	
Full-time (> 30 h/week)	1032 (70.11)
Part-time	165 (11.21)
Homemaker	159 (10.80)
Other	79 (7.32)
***Child and youth demographic profile***	
Age, M (SD) / *n* (%)	
5–11 years	8.12 (2.04) / 693 (47.1)
12–17 years	14.85 (1.68) / 779 (52.9)
Gender female, *n* (%)	775 (52.6)
Disability, *n* (%)	127 (8.6)
Household makeup, M (SD)	
Adults	2.10 (0.67)
Children	1.81 (0.84)
Child’s residence type, *n* (%)	
House	1062 (72.1)
Apartment/Townhouse	392 (26.7)
Other	18 (1.2)
Dog ownership, *n* (%)	521 (35.4)
***Parental assessed child and youth movement behaviours***	
Meeting guidelines, *n* (%)	
Moderate to vigourous physical activity	268 (18.21)
Sleep (5–13 years) / Sleep (14–17 years)	613 (67.80) / 433 (76.20)
Screen time	166 (11.30)
24-h combined	39 (2.60)

### Children and youth’s current behaviours and changes in behaviours

**Fig. 1:**
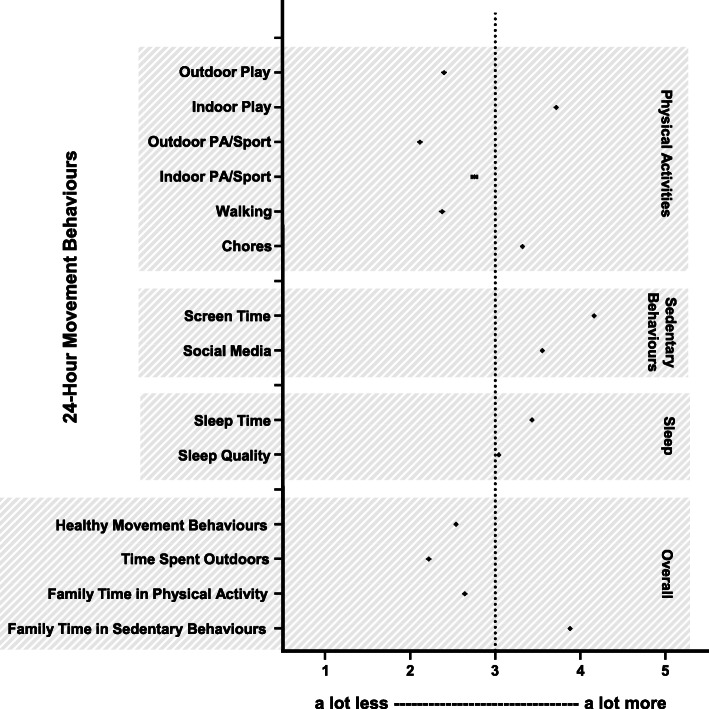
Forest plot of parent-reported changes in 24-h movement behaviours of Canadian children and youth. Forest plot of parent-reported changes in 24-h movement behaviours of Canadian children and youth (5–17 years) based on responses to a 5-point scale ranging from “a lot less” (scored 1) to “about the same” (scored 3) to “a lot more” (scored 5). PA = physical activity. See Table 1 and Additional File 1 for more details on the questions and response options. Data points are means with standard error bars (generally too small to be seen)

A summary of the current movement behaviours of the child and changes in movement and play behaviours of the child by age category and gender are presented in Table 3 and a summary in Fig. 1. More children (23.8%) were meeting the physical activity recommendations compared with youth (13.2%). Children engaged in less leisure screen time (5.1 h/day) compared with youth (6.3 h/day). Very few children and youth were meeting the combined 24-h guidelines (4.8% of children, 0.6% of youth). Fewer girls aged 5–11 years were engaging in sufficient physical activity compared with boys the same age (19.0% girls, 27.9% boys).

**Table 3:** Summary of the movement and play behaviours in children and youth during the COVID-19 virus outbreak

	Children			Youth		
	Total (***n*** = 690)	Girls (***n*** = 321)	Boys (***n*** = 369)	Total (***n*** = 774)	Girls (***n*** = 368)	Boys (***n*** = 406)
**Current child health behaviours, M (SD)**						
MVPA ≥60 min (days/week)	3.55 (2.33)	3.35^a^ (2.26)	3.73^a^ (2.38)	2.59 (2.33)	2.60 (2.28)	2.57 (2.38)
Sleep (hours/day)	9.19 (2.33)	9.28 (2.18)	9.12 (2.45)	9.01 (2.30)	9.10 (2.10)	8.93 (2.46)
Screen time (hours/day)	5.14 (3.54)	5.06 (3.26)	5.21 (3.78)	6.53 (3.31)	6.31^a^ (3.21)	6.72^a^ (3.39)
**Proportion of children meeting guidelines (%)**						
MVPA	23.8	19.0^a^	27.9^ga^	13.2	11.4	14.8
Sleep†	69.9	72.6	67.5	72.1	73.6	70.7
Screen time	16.5	16.2^a^	16.8^a^	6.6	7.9	5.4
24 h combined	4.8	2.8	6.5^ga^	0.6	0.8	0.5
**Change in child movement and play behaviours during COVID-19 outbreak, M (SD)**^a^						
Walks or bikes in neighbourhood	2.57 (1.35)	2.54^a^ (1.36)	2.61^a^ (1.34)	2.19 (1.20)	2.24 (1.24)	2.15 (1.17)
Physical activity or sport outside	2.28 (1.22)	2.26^a^ (1.25)	2.30^a^ (1.20)	1.96 (1.13)	1.93 (1.11)	1.99 (1.15)
Physical activity or sport inside	2.94 (1.15)	3.01^a^ (1.19)	2.88^a^ (1.11)	2.59 (1.21)	2.64 (1.55)	2.55 (1.15)
Household chores	3.35 (0.80)	3.38 (0.83)	3.33 (0.78)	3.29 (0.84)	3.33 (0.81)	3.25 (0.86)
Plays outside	2.58 (1.31)	2.57^a^ (1.30)	2.59^a^ (1.31)	2.24 (1.08)	2.20 (1.03)	2.27 (1.13)
Plays inside	3.85 (1.00)	3.86^a^ (1.02)	3.84^a^ (0.99)	3.60 (1.00)	3.58 (0.97)	3.62 (1.03)
Watches television (TV) or screens	4.10 (0.87)	4.10 (0.92)	4.11 (0.84)	4.21 (0.92)	4.21 (0.91)	4.21 (0.93)
Uses social media	3.30 (0.89)	3.46^g^ (0.91)	3.16 (0.85)	3.78 (0.94)	3.97^ag^ (0.86)	3.62^a^ (0.97)
Sleep quantity	3.21 (0.70)	3.30^g^ (0.70)	3.14 (0.69)	3.63 (0.84)	3.74^ag^ (0.78)	3.53^a^ (0.88)
Sleep quality	3.05 (0.66)	3.05 (0.68)	3.05 (0.65)	3.04 (0.73)	3.05 (0.71)	3.02 (0.75)
Overall healthy movement behaviours	2.66 (0.83)	2.68^a^ (0.81)	2.65^a^ (0.84)	2.44 (0.90)	2.49 (0.91)	2.38 (0.89)
Overall time spent outside	2.38 (1.26)	2.35^a^ (1.25)	2.41^a^ (1.28)	2.08 (1.15)	2.05 (1.13)	2.11 (1.17)
Family time in physical activity	2.72 (1.16)	2.70 (1.17)	2.74 (1.16)	2.57 (1.11)	2.58 (1.06)	2.56 (1.15)
Family time in sedentary behaviours	3.87 (0.81)	3.90 (0.83)	3.85 (0.79)	3.88 (0.88)	3.94 (0.82)	3.83 (0.93)

*MVPA* Moderate to vigorous physical activity; ^a^ = significant age effect. ^g^ = significant gender effect; † = for sleep, the column ‘children’ age is 5–13 years and ‘youth’ age is 14–17 years as per the 24-h sleep guidelines

^a^Range from 1 to 5, 3 represents no change (see Table 1 for details)

Children and youth experienced a significant decline in all physical activities (Table 3), except household chores. The most dramatic decline was with outdoor physical activity and sport (2.28/5.00 and 1.96/5.00 for children and youth, respectively), whereas leisure screen time and social media use was reported as much higher than before the COVID-19 outbreak (leisure screen time 4.10/5.00 and 4.21/5.00 for children and youth respectively; social media 3.30/5.00 and 3.78/5.00 for children and youth, respectively). Parents of girls in both age groups reported significantly more social media use (child: 3.46/5.00 [girls] versus 3.16/5.00 [boys]; youth: 3.97/5.00 [girls] versus 3.62/5.00 [boys]) and more time sleeping (child: 3.30/5.00 [girls] versus 3.14/5.00 [boys]; youth: 3.74/5.00 [girls] versus 3.53/5.00 [boys]) as a result of COVID-19 compared with boys. Respondents reported more family time was being spent in sedentary behaviours (3.87/5.00 and 3.88/5.00 in children and youth, respectively) and less in physical activities (2.72/5.00 and 2.57/5.00 in children and youth, respectively) compared with before the COVID-19 outbreak.

### Parent support of child movement and play behaviours

A complete description of the associations between perceived changes in child movement behaviours and parental demographic, social, and environmental factors are reported in Table 4. Only selected associations (*r* > 0.1, *p* < 0.01) [21] are highlighted here. Being a younger parent was associated with less decline in child physical activity (− 0.11), outdoor (− 0.16) and indoor play (− 0.10), family physical activity (− 0.11), less social media use (− 0.13) and more sleep (0.12). Parental marital status (cohabitation) was correlated with more child outdoor play (0.10) and household chores (0.10). Children that lived in detached homes had more outside physical activity (0.12) and spent more time walking and biking (0.13). Having a dog was associated with more outdoor play (0.11) and less indoor play (− 0.10). Parental encouragement was associated with higher child outdoor physical activity (0.17), time spent walking and biking (0.12), outdoor play (0.14), indoor physical activity (0.12), and family physical activity (0.24). Parent co-participating was associated with more child outdoor physical activity (0.32), time spent walking and biking (0.32), outdoor play (0.39), indoor physical activity (0.14), household chores (0.18), and family physical activity (0.44).

**Table 4:** Associations between perceived changes in movement behaviours and demographic, social, and environmental factors

	Outside physical activity	Walking and biking	Outside play	Overall time outdoors	Inside physical activity	Household chores	Inside play	Family physical activity	Family sedentary behaviour	Screen time	Social media	Sleep	Sleep quality
Parent’s age	−0.11*	−0.11*	−0.16*	−0.14*	−0.10*	−0.02	−0.07*	−0.11*	−0.01	0.04	−0.13*	0.12*	−0.07
Parent’s gender (1 = M; 2 = F)	−0.01	0.00	0.04	0.02	−0.02	0.06	0.08*	0.03	0.05	0.08*	0.08*	0.07*	0.01
Parent’s education	− 0.05	0.02	− 0.06	− 0.07*	− 0.01	− 0.01	0.02	− 0.05	0.09*	0.03	0.09*	− 0.01	0.03
Parent’s work status (1 = U; 2 = E)	0.02	0.04	0.01	0.03	0.02	−0.02	− 0.02	0.04	0.03	−0.03	0.03	0.01	0.04
Household income	0.03	0.13*	0.11*	0.05	0.01	0.07*	−0.03	0.04	0.07*	0.04	0.11*	0.01	0.02
Marital status^a^	0.06	0.06	0.11*	0.08*	0.05	0.10*	0.01	0.08*	−0.02	−0.05	−0.06	− 0.05	0.05
Household type^b^	0.12*	0.13*	0.18*	0.14*	−0.01	0.09*	−0.09*	0.09*	0.01	0.01	0.04	−0.04	−0.03
Dog ownership (1 = no; 2 = yes)	0.07*	0.03	0.11*	0.10*	0.00	0.09*	−0.10*	0.06	−0.02	− 0.01	0.09*	0.07*	0.02
Parent encourages physical activity	0.17*	0.12*	0.14*	0.13*	0.12*	0.08*	−0.02	0.24*	0.05	0.07*	0.04	0.10*	0.07*
Parent participates in physical activity	0.32*	0.32*	0.39*	0.39*	0.14*	0.18*	−0.10*	0.44*	0.04	−0.02	0.00	−0.02	0.04
Parent supports physical activity	0.14*	0.10*	0.12*	0.15*	0.09*	−0.01	−0.10*	0.13*	−0.10	− 0.07*	−0.03	− 0.03	−0.02
Parent discourages screen time	−0.06	−0.05	− 0.01	−0.06	0.00	0.08*	0.11*	0.00	0.06	0.13*	0.09*	0.09*	0.00
Parent encourages sleep	0.00	−0.03	−0.07*	−0.04	0.05	0.09*	0.13*	−0.01	0.07*	0.06	0.04	0.16*	0.15*

* = *p* < 0.01; *M* Male, *F* Female, *U* Unemployed, *E* Employed, ^a^Marital status, 1 = single, 2 = cohabited; ^b^Household type, 1 = other, 2 = detached house

### New ways families are approaching movement behaviours

Half of respondents (50.4%) indicated their child was doing a lot more inside hobbies or activities, and 22.7% reported increased outside hobbies or activities. Among those that provided a written response, the top three increased inside activities were arts and crafts (12.9%), puzzles and games (11.3%), and video games (10.2%). Among the reported increased inside activities, 17.5% were screen-based (e.g., phone, tablet, television) while 2.6% were active hobbies (e.g., dancing, physical education exercises, treadmill). The top three increased outside activities were biking (6.1%), walking or hiking (5.5%) and sport activities (3.5%; e.g., badminton, basketball on driveway, road hockey). New family hobbies as a result of the COVID-19 outbreak were also reported (273 responses), with the most common new activities including puzzles and games (7.1%), arts and crafts (4.4%), and physical activity (1.0%). Among respondents, 16.4% reported using online resources or apps to support healthy movement behaviours. Complete details of open-ended questions are provided in Additional File 3.

## Discussion

The purpose of this study was to assess the immediate changes in physical activity, play, sedentary behaviours, leisure screen time, and sleep in school-aged children and youth across Canada during the initial period of the COVID-19 crisis. We found that children and youth were less active, played outside less, were more sedentary, engaged in more recreational screen-based activities, and slept more during the initial COVID-19 virus outbreak compared with before the restrictions. We observed commonly found gender and age group related differences [22]**.** In general, girls were less active than boys and youth (12–17 years) were less active than children (5–11 years). Girls engaged in more social media use and slept more than boys. The largest reported change in behaviours related to leisure screen-based activities, where children and youth were watching upwards of 6.5 h per day. On average, younger children experienced less change from their pre-COVID-19 movement behaviours compared with older children. These findings are the first to confirm speculations that pandemic-related restrictions are unfavourably related to movement behaviours of children and youth [13, 23]**.** This observation has triangulated support from quantitative (descriptive and correlational) and qualitative (contextual) evidence.

The prevalence of children and youth meeting the Canadian 24-Hour Movement Behaviour Guidelines [3] was much lower in this sample (2.6%, Table 2) compared to other national samples collected before the COVID-19 outbreak. For example, based on similar data collection methods, Rhodes et al. (2019) reported 12.7% of Canadian 5–17 year-olds met the guideline [5]. Using more robust measures, including accelerometer-measured physical activity, Carson et al. (2017) reported 17.1% Canadian children and youth (5–17 years) met the overall guidelines [1]. An international sample of 9–11 year-old children from 12 countries reported an average of 7.2% (Canada 14.0%) meeting the guidelines in 2016 before the COVID-19 virus outbreak [24]. These comparisons suggest a dramatic reduction in the proportion of Canadian children and youth meeting these guidelines during the COVID-19 outbreak.

The reported reduction in outdoor play may contribute to or even exacerbate the decline reported in physical activity. Correlations between moderate-to-vigorous *(r* = 0.34; *p* < 0.01) and light physical activity (*r* = 0.28; *p* < 0.01) and changes in outdoor time supports this point. Spending time outdoors is associated with greater physical activity, less sedentary time, improved sleep, and a number of other benefits (e.g., mental health, immune function) [6, 7]. Active play indoors does not seem to replace active play outdoors resulting in a net decline in reported play-based activity. To prevent the unintended unhealthy behaviour consequences of COVID-19 restrictions and ‘stay home’ advice, health promotion messaging needs to be balanced with disease prevention messaging [25]. With attentive and responsible spatial and temporal distancing a healthy marriage of “stay home” and “get outside and play” is achievable.

We identified several factors that helped to support physical activity, outdoor play, and sleep, and reduce time spent in screen-based and other sedentary behaviours. This highlights that the impact of the pandemic has not been uniform for all Canadian children and youth. Living in a detached house, being a younger parent, and owning a dog were all favourably associated with healthy movement behaviours. Children living in a house versus an apartment may have easier access to front or back yards for outdoor play and physical activity [26]**.** The largest associations were noted with parental encouragement for and engagement in healthy movement behaviours. Similar to previous research [5, 19, 27]**,** we found that parent support was a key correlate of children and youth movement behaviours. The most significant relationship was parental co-participation [27]**.** Although parents and their children are undoubtedly experiencing higher stress during the COVID-19 outbreak [28]**,** promoting physical activity and outdoor play by targeting co-participation, while respecting public health restrictions, may be one strategy to enhance health behaviours of children and youth [28, 29]**.** The associations observed with parental age are consistent with typical age-related declines in physical activity [29]**.** Finally, families who had a dog had higher physical activity and outdoor time. A recent systematic review also showed that dog-related interventions increased physical activity [30]**.**

There are emerging calls bringing attention to the likely impact of the global pandemic on the movement behaviours of adults, children and youth [31, 32]. Our findings describe some of the ways Canadian parents are adapting to this challenge. In the open-ended questions, respondents of this survey identified several creative ways that they were using their leisure-time to develop or renew new family hobbies. Families are reconnecting through leisure, though primarily sedentary leisure activities. Carving out time for family leisure and starting new hobbies and activities may be helpful strategies to reduce the mental health challenges (e.g., depression, anxiety) that are being exacerbated by lockdown conditions [33, 34]. Where possible, families should consider substituting sedentary leisure for more active leisure pursuits. A recent review of reviews demonstrated that increased physical activity was associated with decreased depressive symptoms in children and youth [35] and suggested that physical activity should be included in interventions to reduce the public health burden of mental illness. Many children and their families may characterize the COVID-19 virus outbreak and lockdown as traumatic [36]. Adopting healthy movement behaviours may help to mitigate the negative effects on children and youth of this pandemic [13].

Acknowledging the challenges in meeting movement behaviour guidelines and based on the advice of parents surveyed in this study, we recommend that:

1. Parents continue to be creative in their home-based leisure activities and support and encourage their children to play and be active in innovative and safe ways. Suggestions include co-participation in activities, trying new leisure hobbies, using online health and/or physical activity apps, and getting outdoors as much as possible (while following public health requirements). It is recommended that children and youth aim to accumulate 60 min per day of moderate-to-vigorous physical activity and play outdoors regularly [3, 4, 6, 7].
2. Parents continue to set routines for their children, including supervised time for screens, regular sleep and wake times, and time for quality family time. Limit leisure screen time to 2 h per day and swap screen time for play time wherever possible [3, 4].
3. Public health officials support parents by implementing safe physical distancing measures that provide extra space for everyone to walk, cycle, wheel, and scoot. This could include temporary reallocation of roadway space and keeping expansive green spaces open.

### Strengths, limitations, and future directions

Given that the evidence on the effect of the COVID-19 virus outbreak on children’s health-related behaviours is scarce, this study has many of strengths. First, the sample was a nationally representative cohort of over 1500 parents of school-aged children and youth. Second, we assessed parental-related factors to determine associations between parental behaviours (e.g., co-play) and child and youth behaviours. Our study was limited by its cross-sectional design although given our aim this approach was appropriate. The parent-report nature of the study and the possibility of social desirability and/or recall bias may affect our findings. While we were able to assess associations between parent work status and child movement and play behaviours, we did not have data to indicate whether or not parents were working from home or had lost their job as a result of the pandemic. Further, sampled parents represented the geographic, cultural, and socioeconomic make up of Canada, however it should be noted that parents received a small financial incentive for participation in each survey (up to $3.00). Results may not generalize to other countries. Future studies should evaluate the longer-term consequences of the COVID-19 virus outbreak and recovery on the movement behaviours of children and youth. To develop targeted health promotion strategies [25], it would be useful to identify province-specific or geographic differences influencing health behaviours of children and youth.

## Conclusion

This study provides evidence of immediate collateral consequences of the COVID-19 outbreak, demonstrating an adverse impact on the movement and play behaviours of Canadian children and youth. The findings highlight the challenge of, but need for, a balance of disease prevention and health promotion efforts specific to integrative movement behaviours. Accordingly, these findings can guide efforts to preserve and promote child health during the COVID-19 outbreak and crisis recovery period, and inform strategies to mitigate potential harm during future pandemics.

## Supplementary information

**Additional file 1:.** Complete children and youth movement and play behaviours survey items.

**Additional file 2:.** STROBE statement checklist of items for cross-sectional studies.

**Additional file 3: Table 1.** Inside and outside hobbies during COVID-19 virus outbreak. **Table 2.** New or returning family hobbies during the COVID-19 virus outbreak. **Table 3**. Use of online resources during the COVID-19 virus outbreak.

## Data Availability

The dataset supporting the conclusion of this article is available from ParticipACTION upon reasonable request and the completion of a data transfer agreement.
