# Supplementary material for: Impact of the COVID-19 virus outbreak on movement and play behaviours of Canadian children and youth: a national survey
Source: Int J Behav Nutr Phys Act. 2020 Jul 6;17:85. doi: 10.1186/s12966-020-00987-8 (PMC7336091; doi:10.1186/s12966-020-00987-8)
Supplement: Supplementary file 1 — Additional file 1:. Complete children and youth movement and play behaviours survey items. [file 12966_2020_987_MOESM1_ESM.docx]

**Additional File 1.** Complete children and youth movement and play behaviours survey items


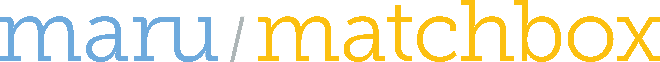


**CHILDREN & YOUTH MOVEMENT AND PLAY BEHAVIOURS SURVEY:**

**IMPACT OF THE 2020 COVID-19 OUTBREAK**

Final Questionnaire: April 9, 2020

**SURVEY DETAILS**

**Survey Respondent Details:**

1. Quote Code: Add quote code
2. Languages: English
3. Device Type: Desktop + Mobile

**Standard Demographics:**

Below are the standard demographics that are automatically included in projects using Maru/Blue sample. Click on each demographic to preview the question text, answer options and standard rollups where available:

[Age](https://surveytest.ca.matchbox.maruhub.com/index/enter/s/ESV-vlt1-471927167/case/777888369) *(incl. Age Rollup)*

[Gender](https://surveytest.ca.matchbox.maruhub.com/index/enter/s/ESV-vlt1-471927167/case/672649679)

[Canadian Regions](https://surveytest.ca.matchbox.maruhub.com/index/enter/s/ESV-vlt1-471927167/case/592383229) *(incl. Postal Code, Province, SAC*)

[US Regions](https://surveytest.ca.matchbox.maruhub.com/index/enter/s/ESV-vlt1-471927167/case/592383229) *(incl. Zipcode, Division, 4 Region Rollup, DMA, FIPS)*

[Education](https://surveytest.ca.matchbox.maruhub.com/index/enter/s/ESV-vlt1-471927167/case/925138302)

[Employment](https://surveytest.ca.matchbox.maruhub.com/index/enter/s/ESV-vlt1-471927167/case/763750843)

[Marital Status](https://surveytest.ca.matchbox.maruhub.com/index/enter/s/ESV-vlt1-471927167/case/278673460)

[Ethnicity](https://surveytest.ca.matchbox.maruhub.com/index/enter/s/ESV-vlt1-471927167/case/213006247) (incl. Hispanic/Latino in US)

[Household Income](https://surveytest.ca.matchbox.maruhub.com/index/enter/s/ESV-vlt1-471927167/case/172800273)

[Children in Household](https://surveytest.ca.matchbox.maruhub.com/index/enter/s/ESV-vlt1-471927167/case/982665676)

**Study Timelines:**

Below are the study milestones and associated timelines:

| MILESTONE | DATE |
| --- | --- |
| Draft Questionnaire Delivered: | Click to select a date. |
| Final Questionnaire Approved: | April 9, 2020 |
| Programming + QA: | April 9, 2020 **to**  April 15, 2020 |
| Fielding: | April 15, 2020 **to** April 20, 2020 |
| Deliverables: | April 27, 2020 |

**Survey Quotas:**

Below are the entry and in-study quota details:

| QUOTA | CRITERIA | AMOUNT | HARD / SOFT | MIN / MAX |
| --- | --- | --- | --- | --- |
| Entry Quota | Total Respondents |  |  |  |
| Sample flag 1 | Children aged 5-11 | 750 |  |  |
| Sample flag 2 | Children aged 12-17 | 750 |  |  |
|  |  |  |  |  |

**Termination Text:**

*If your study is using IC or Client Supplied sample, please specify termination text below or delete this section if not applicable.*

| TERMINATION TYPE | TEXT | REDIRECT (if applicable) |
| --- | --- | --- |
| Disqualification |  |  |
| Over Quota |  |  |
| Complete |  |  |

| **SCREENER** |
| --- |

**[QUESTION TYPE:** single choice**] [LAYOUT:** default (kato)**]**

**[QCOVID_SCREEN] [REQUIRED]**

**Base: Total**

Has **anyone in your household** been diagnosed with COVID-19?

**[ANSWERS] [ANCHOR]**

Yes [THANK AND TERMINATE]

No

TAB NOTE:

Detail Table Requirements

**[QUESTION TYPE:** single choice**] [LAYOUT:** default (kato)**]**

**[QQUARANTINE] [REQUIRED]**

**Base: Total**

Is your household under a self-isolation or quarantine order (i.e., not allowed to leave the house, due to travel or other potential exposure to COVID-19)?

**[ANSWERS] [ANCHOR]**

Yes [THANK AND TERMINATE]

No

TAB NOTE:

Detail Table Requirements

| **DEMOGRAPHICS** |
| --- |

**[QUESTION TYPE:** text instruction**]**

**[QINSTRUCTION] [REQUIRED]**

**Base: Total**

The first set of questions ask about your child’s movement behaviours in the last week. If you have more than one child who is aged **[INSERT AGE RANGE BASED ON SAMPLE FLAG; EITHER 5-11 OR 12-17]**, please think about the child who’s name is first alphabetically

**[QUESTION TYPE:** open end**] [LAYOUT:** text field (single line) narrowtextbox - numeric (kato)**]**

**[QChildAge] [REQUIRED]**

**Base: Total**

How old is your child that you will be answering the survey about?

TAB NOTE:

Detail Table Requirements

**[QUESTION TYPE:** single choice**] [LAYOUT:** default (kato)**]**

**[QGender] [REQUIRED]**

**Base: Total**

What gender does your child that you will be answering the survey about identify as?

**[ANSWERS] [ANCHOR]**

Male

Female

They identify as [SPECIFY]

I’d rather not say

TAB NOTE:

Detail Table Requirements

**[QUESTION TYPE:** single choice**] [LAYOUT:** default (kato)**]**

**[QDisabilitiy] [REQUIRED]**

**Base: Total**

Does your child have a diagnosed disability or chronic condition?

**[ANSWERS] [ANCHOR]**

Yes [specify]

No

TAB NOTE:

Detail Table Requirements

**[QUESTION TYPE:** open end**] [LAYOUT:** text field (single line) default (kato)**]**

**[QPostal Code] [REQUIRED]**

**Base: Total**

What are the first three digits of the postal code of the primary residence of your child that you will be answering the survey about?

TAB NOTE:

Detail Table Requirements

**[QUESTION TYPE:** single choice**] [LAYOUT:** default (kato)**]**

**[Qdwellingtype] [REQUIRED]**

**Base: Total**

And what type of home is the primary residence of your child who you will be answering the survey about?

**[ANSWERS] [ANCHOR]**

Low rise Apartment/Condo

High rise Apartment/Condo

Townhouse

Semi-detached house

Detached house

Something else [SPECIFY]

TAB NOTE:

Detail Table Requirements

**[QUESTION TYPE:** open end**] [LAYOUT:** text field (single line) narrowtextbox - numeric (kato)**]**

**[QHHComp] [REQUIRED]**

**Base: Total**

And what is the household makeup of your child’s primary residence?

______ adults

______children

TAB NOTE:

Detail Table Requirements

**[QUESTION TYPE:** single choice**] [LAYOUT:** default (kato)**]**

**[QDog] [REQUIRED]**

**Base: Total**

Does your family have a dog or dogs that require regular walking/exercise?

**[ANSWERS] [ANCHOR]**

Yes

No

TAB NOTE:

Detail Table Requirements

| **CURRENT MOVEMENT BEHAVIOURS** |
| --- |

**[QUESTION TYPE:** open end**] [LAYOUT:** text field (single line) narrowtextbox - numeric (kato)**]**

**[QScreenTimeAvg] [REQUIRED]**

**Base: Total**

On average, how many total hours and minutes per day did your child watch TV, use the computer, use social media and inactive play video games, during their free time over the last week?

Weekdays (per day) _______ Hours (Cap at 23) AND _______ Minutes (Cap at 59) – Don’t know

Weekend (per day) _______ Hours (Cap at 23) AND _______ Minutes (Cap at 59) – Don’t know

TAB NOTE:

Detail Table Requirements

**[QUESTION TYPE:** text instruction**]**

**[QMVPADef] [REQUIRED]**

**Base: Total**

The next questions relate to physical activity…

Moderate-to-vigorous physical activity is any activity that increases your heart rate and makes you get out of breath some of the time. Examples include sports, school activities, playing with friends, or walking to school.

**[DROP DOWN]**

**Q27**

**Base = Total**

**In the last week, on how many days did your child engage in moderate-to-vigorous physical active for a total of at least 60 minutes per day?**

Drop down <<< 0-7 days >>>

**[DROP DOWN]**

**Q28**

**Base = Total**

**Light physical activity is physical activity that does not result in sweat production or shortness of breath. Examples include mild stretching, playing with animals, and leisurely walking.**

**In the last week, on how many days did your child engage in light physical activity for 2 or more hours per day?**

Drop down <<<0-7 days>>>

**[DROP DOWN]**

**Q29**

**Base = Total**

**In the last week, how many hours did your child usually spend sleeping in a 24-hour period (including naps but excluding time spent resting)?**

Dropdown <<<0-24 hours>>>

| **CHANGE IN MOVEMENT AND PLAY BEHAVIOURS** |
| --- |

**[QUESTION TYPE:** text instruction**]**

**[QWHO] [REQUIRED]**

**Base: Total**

***The World Health Organization (WHO) declared COVID-19 as a Pandemic on March 11, 2020. For the following questions, we would like you to consider how your child’s behaviours (physical activity, play, sedentary behaviours, digital screen use, and sleep) changed as a result of COVID-19 and related restrictions.***

**[QUESTION TYPE:** single choice**] [LAYOUT:** default (kato)**]**

**[QPre_Walks] [REQUIRED]**

**Base: Total**

**Compared to before the COVID-19 outbreak and related restrictions**, my child walks or bikes in the neighbourhood?

**[ANSWERS] [ANCHOR]**

A lot less

A little less

About the same

A little more

A lot more

TAB NOTE:

Detail Table Requirements

**[QUESTION TYPE:** single choice**] [LAYOUT:** default (kato)**]**

**[QPre_SportsOUT] [REQUIRED]**

**Base: Total**

**Compared to before the COVID-19 outbreak and related restrictions**, my child is doing physical activities or sport outside?

**[ANSWERS] [ANCHOR]**

A lot less

A little less

About the same

A little more

A lot more

TAB NOTE:

Detail Table Requirements

**[QUESTION TYPE:** single choice**] [LAYOUT:** default (kato)**]**

**[QPre_SportsIN] [REQUIRED]**

**Base: Total**

**Compared to before the COVID-19 outbreak and related restrictions**, my child is doing physical activities or sport inside?

**[ANSWERS] [ANCHOR]**

A lot less

A little less

About the same

A little more

A lot more

TAB NOTE:

Detail Table Requirements

**[QUESTION TYPE:** single choice**] [LAYOUT:** default (kato)**]**

**[QPre_Chores] [REQUIRED]**

**Base: Total**

**Compared to before the COVID-19 outbreak and related restrictions**, my child is doing household chores (e.g. cleaning, yard work)?

**[ANSWERS] [ANCHOR]**

A lot less

A little less

About the same

A little more

A lot more

TAB NOTE:

Detail Table Requirements

**[QUESTION TYPE:** single choice**] [LAYOUT:** default (kato)**]**

**[QPre_PlaysOUT] [REQUIRED]**

**Base: Total**

**Compared to before the COVID-19 outbreak and related restrictions**, my child plays outside?

**[ANSWERS] [ANCHOR]**

A lot less

A little less

About the same

A little more

A lot more

TAB NOTE:

Detail Table Requirements

**[QUESTION TYPE:** multi choice**] [LAYOUT:** default (kato)**]**

**[QCommonPlaces] [REQUIRED]**

**Base: Total**

If your child is spending any time outside during the COVID-19 outbreak, where are the common places this outside time is being spent?

**[ANSWERS] [RANDOMIZE]**

Yard or driveway

Sidewalks, parking lots, or neighbourhood streets

Parks within walkable distance

Trails within walkable distance

School grounds or outside spaces within walkable distance

Parks where you have to drive

Trails where you have to drive

School grounds or outside spaces where you have to drive

Other **[SPECIFY][ANCHOR]**

Not Applicable **[ANCHOR][EXCLUSIVE]**

TAB NOTE:

Detail Table Requirements

**[QUESTION TYPE:** single choice**] [LAYOUT:** default (kato)**]**

**[QPlays_Inside] [REQUIRED]**

**Base: Total**

Compared to before the COVID-19 outbreak and related restrictions, my child plays inside?

**[ANSWERS] [ANCHOR]**

A lot less

A little less

About the same

A little more

A lot more

TAB NOTE:

Detail Table Requirements

**[QUESTION TYPE:** single choice**] [LAYOUT:** default (kato)**]**

**[QPrePlaymate] [REQUIRED]**

**Base: Total**

BEFORE the COVID-19 outbreak and related restrictions, my child’s primary playmate was?

**[ANSWERS] [RANDOMIZE]**

Themselves

Sibling(s)

Parent

Other Caregiver

Friend(s)

Other – Specify [ANCHOR]

TAB NOTE:

Detail Table Requirements

**[QUESTION TYPE:** single choice**] [LAYOUT:** default (kato)**]**

**[QPostPlaymate] [REQUIRED]**

**Base: Total**

Now, my child’s primary playmate is?

**[ANSWERS] [RANDOMIZE]**

Themselves

Sibling(s)

Parent

Other Caregiver

Friend(s)

Other – Specify [ANCHOR]

TAB NOTE:

Detail Table Requirements

**[QUESTION TYPE:** single choice**] [LAYOUT:** default (kato)**]**

**[QScreenTime] [REQUIRED]**

**Base: Total**

Compared to before the COVID-19 outbreak and related restrictions, my child watches TV, movies, uses the computer for leisure or plays sedentary video games?

**[ANSWERS] [ANCHOR]**

A lot less

A little less

About the same

A little more

A lot more

TAB NOTE:

Detail Table Requirements

**[QUESTION TYPE:** single choice**] [LAYOUT:** default (kato)**]**

**[QSocialMedia] [REQUIRED]**

**Base: Total**

Compared to before the COVID-19 outbreak and related restrictions, my child uses social media?

**[ANSWERS] [ANCHOR]**

A lot less

A little less

About the same

A little more

A lot more

TAB NOTE:

Detail Table Requirements

**[QUESTION TYPE:** single choice**] [LAYOUT:** default (kato)**]**

**[QSedentary] [REQUIRED]**

**Base: Total**

Compared to before the COVID-19 outbreak and related restrictions, does other sedentary leisure activities not in front of screens (e.g., reading, puzzles, crafts, music or art)?

**[ANSWERS] [ANCHOR]**

A lot less

A little less

About the same

A little more

A lot more

TAB NOTE:

Detail Table Requirements

**[QUESTION TYPE:** single choice**] [LAYOUT:** default (kato)**]**

**[QSleep] [REQUIRED]**

**Base: Total**

Compared to before the COVID-19 outbreak and related restrictions, my child sleeps?

**[ANSWERS] [ANCHOR]**

A lot less

A little less

About the same

A little more

A lot more

TAB NOTE:

Detail Table Requirements

**[QUESTION TYPE:** single choice**] [LAYOUT:** default (kato)**]**

**[QSleepQuality] [REQUIRED]**

**Base: Total**

Compared to before the COVID-19 outbreak and related restrictions, my child’s sleep quality is?

**[ANSWERS] [ANCHOR]**

A lot worse

A little worse

About the same

A little better

A lot better

TAB NOTE:

Detail Table Requirements

**[QUESTION TYPE:** single choice**] [LAYOUT:** default (kato)**]**

**[QOverallQuality] [REQUIRED]**

**Base: Total**

Compared to before the COVID-19 outbreak and related restrictions, the balance of my child’s overall healthy movement behaviours (i.e., physical activity, sedentary behaviours, and sleep) are?

**[ANSWERS] [ANCHOR]**

A lot worse

A little worse

About the same

A little better

A lot better

TAB NOTE:

Detail Table Requirements

**[QUESTION TYPE:** single choice**] [LAYOUT:** default (kato)**]**

**[QTotalOutside] [REQUIRED]**

**Base: Total**

Compared to before the COVID-19 outbreak and related restrictions, my child’s overall time spent outside is?

**[ANSWERS] [ANCHOR]**

A lot less

A little less

About the same

A little more

A lot more

TAB NOTE:

Detail Table Requirements

**[QUESTION TYPE:** single choice**] [LAYOUT:** default (kato)**]**

**[QTotalOutsideGeneral] [REQUIRED]**

**Base: Total**

Compared to before the COVID-19 outbreak and related restrictions, I see children playing outside?

**[ANSWERS] [ANCHOR]**

A lot less

A little less

About the same

A little more

A lot more

TAB NOTE:

Detail Table Requirements

**[QUESTION TYPE:** single choice**] [LAYOUT:** default (kato)**]**

**[QTotalOutside] [REQUIRED]**

**Base: Total**

Compared to before the COVID-19 outbreak and related restrictions, our family time spent in physical activity is?

**[ANSWERS] [ANCHOR]**

A lot less

A little less

About the same

A little more

A lot more

TAB NOTE:

Detail Table Requirements

**[QUESTION TYPE:** single choice**] [LAYOUT:** default (kato)**]**

**[QTotalOutside] [REQUIRED]**

**Base: Total**

Compared to before the COVID-19 outbreak and related restrictions, our family time spent in sedentary behaviours (e.g., watching TV, playing board games, doing crafts) is?

**[ANSWERS] [ANCHOR]**

A lot less

A little less

About the same

A little more

A lot more

TAB NOTE:

Detail Table Requirements

**[QUESTION TYPE:** single choice**] [LAYOUT:** default (kato)**]**

**[QInsideHobbyMore] [REQUIRED]**

**Base: Total**

As a result of the COVID-19 outbreak and related restrictions, is there an inside leisure activity or hobby that your child is doing a lot more now?

**[ANSWERS] [ANCHOR]**

Yes **[SPECIFY]**

No

TAB NOTE:

Detail Table Requirements

**[QUESTION TYPE:** single choice**] [LAYOUT:** default (kato)**]**

**[QOutsideHobbyMore] [REQUIRED]**

**Base: Total**

As a result of the COVID-19 outbreak and related restrictions, is there an outside leisure activity or hobby that your child is doing a lot more now?

**[ANSWERS] [ANCHOR]**

Yes **[SPECIFY]**

No

TAB NOTE:

Detail Table Requirements

**[QUESTION TYPE:** single choice**] [LAYOUT:** default (kato)**]**

**[QFamilyHobbyMore] [REQUIRED]**

**Base: Total**

As a result of the COVID-19 outbreak and related restrictions, has your family begun any new or novel activities not previously practiced?

**[ANSWERS] [ANCHOR]**

Yes **[SPECIFY]**

No

TAB NOTE:

Detail Table Requirements

**[QUESTION TYPE:** single choice**] [LAYOUT:** default (kato)**]**

**[QOnlineResources] [REQUIRED]**

**Base: Total**

As a result of the COVID-19 outbreak and related restrictions, has your family used any online resources or apps to support healthy movement behaviours?

**[ANSWERS] [ANCHOR]**

Yes **[SPECIFY]**

No

TAB NOTE:

Detail Table Requirements

**[QUESTION TYPE:** single choice**] [LAYOUT:** default (kato)**]**

**[QHealthCondition] [REQUIRED]**

**Base: Total**

As a result of the COVID-19 outbreak and related restrictions, has there been a decrease in your child’s health (e.g., existing condition worsened or new condition developed)?

**[ANSWERS] [ANCHOR]**

Yes **[SPECIFY]**

No

TAB NOTE:

Detail Table Requirements

**[QUESTION TYPE:** single choice**] [LAYOUT:** default (kato)**]**

**[QDistress] [REQUIRED]**

**Base: Total**

Using the scale below, please select the number that best describes how much distress your family has been experiencing over the past week.

**[ANSWERS] [ANCHOR]**

0 (No Distress)

1

2

3

4

5

6

7

8

9

10 (Extreme Distress)

TAB NOTE:

Detail Table Requirements

| **ENCOURAGING CHILD MOVEMENT BEHAVIOURS** |
| --- |

**[QUESTION TYPE:** single choice**] [LAYOUT:** default (kato)**]**

**[QEncourageOutside] [REQUIRED]**

**Base: Total**

Compared to before the COVID-19 outbreak and related restrictions, I have encouraged my child to participate in physical activity or sport?

**[ANSWERS] [ANCHOR]**

A lot less

A little less

About the same

A little more

A lot more

TAB NOTE:

Detail Table Requirements

**[QUESTION TYPE:** single choice**] [LAYOUT:** default (kato)**]**

**[QPlayKidsOutside] [REQUIRED]**

**Base: Total**

Compared to before the COVID-19 outbreak and related restrictions, I play outside with my child or do moderate-to-vigorous physical activity with my child?

**[ANSWERS] [ANCHOR]**

A lot less

A little less

About the same

A little more

A lot more

TAB NOTE:

Detail Table Requirements

**[QUESTION TYPE:** single choice**] [LAYOUT:** default (kato)**]**

**[QEncourageOutside] [REQUIRED]**

**Base: Total**

Compared to before the COVID-19 outbreak and related restrictions, I drove or provided transportation for my child to do physical activity or sport?

**[ANSWERS] [ANCHOR]**

A lot less

A little less

About the same

A little more

A lot more

TAB NOTE:

Detail Table Requirements

**[QUESTION TYPE:** single choice**] [LAYOUT:** default (kato)**]**

**[QEncourageOutside] [REQUIRED]**

**Base: Total**

Compared to before the COVID-19 outbreak and related restrictions, I have encouraged my child to do household chores (e.g., cleaning, yard work)?

**[ANSWERS] [ANCHOR]**

A lot less

A little less

About the same

A little more

A lot more

TAB NOTE:

Detail Table Requirements

**[QUESTION TYPE:** single choice**] [LAYOUT:** default (kato)**]**

**[QDiscourageSedentary] [REQUIRED]**

**Base: Total**

Compared to before the COVID-19 outbreak and related restrictions, I have encouraged my child to stop sitting and watching screens?

**[ANSWERS] [ANCHOR]**

A lot less

A little less

About the same

A little more

A lot more

TAB NOTE:

Detail Table Requirements

**[QUESTION TYPE:** single choice**] [LAYOUT:** default (kato)**]**

**[QEncourageSleep] [REQUIRED]**

**Base: Total**

Compared to before the COVID-19 outbreak and related restrictions, I encouraged my child to sleep between 9-11 hours per night?

**[ANSWERS] [ANCHOR]**

A lot less

A little less

About the same

A little more

A lot more

TAB NOTE:

Detail Table Requirements

**[QUESTION TYPE:** single choice grid**] [LAYOUT:** default (kato)**]**

**[QAgreementBehaviour] [REQUIRED]**

**Base: Total**

Please indicate how much you agree or disagree with each of the following statements.

**Assuming I am fully motivated…**

**[COLUMNS] [ANCHOR]**

Strongly disagree

Disagree

Neutral

Agree

Strongly agree

**[ROWS] [RANDOMIZE]**

I am capable of supporting my child’s physical activity over the next two weeks

I will have an opportunity to support my child’s physical activity over the next two weeks

I am capable of restricting my child’s screen time to no more than 2 hours per day over the next two weeks

I will have an opportunity to restrict my child’s screen time to no more than 2 hours per day over the next two weeks

I am capable of supporting my child’s sleep over the next two weeks

I will have an opportunity to support my child’s sleep over the next two weeks

TAB NOTE:

Detail Table Requirements

**[QUESTION TYPE:** single choice**] [LAYOUT:** default (kato)**]**

**[QHealthCondition] [REQUIRED]**

**Base: Total**

Do you have any advice for families trying to achieve a healthy balance of movement behaviours (physical activity, screen time, sleep) of their children during the COVID-19 outbreak and related restrictions?

**[ANSWERS] [ANCHOR]**

Yes **[SPECIFY]**

No

TAB NOTE:

Detail Table Requirements

| **FOLLOW UP** |
| --- |

**[QUESTION TYPE:** single choice**] [LAYOUT:** default (kato)**]**

**[QFGInterest] [REQUIRED]**

**Base: Total**

We may be conducting online focus groups or additional discussions on this topic. Would you be interested in potentially participating in this research if it were to proceed?

**[ANSWERS] [ANCHOR]**

Yes, I’d be interested

No, I’m not interested

TAB NOTE:

Detail Table Requirements

**[QUESTION TYPE:** open end**] [LAYOUT:** text field (single line) default (kato)**]**

**[QPII] [REQUIRED]**

**Base: IF YES AT QFGINTEREST**

Thank you for your interest! Any information you provide here will be used strictly to contact you for any follow up research related specifically to this survey. You may hear from the sponsors of this survey to recruit you to the follow up research, but your information will never be sold or given to any other third party company.

Name

Email address

Re-enter Email address [EMAIL VALIDATION]

[ADD CHECK BOX] I would rather not participate

TAB NOTE:

Detail Table Requirements
