# Supplementary material for: Impact of the COVID-19 virus outbreak on movement and play behaviours of Canadian children and youth: a national survey
Source: Int J Behav Nutr Phys Act. 2020 Jul 6;17:85. doi: 10.1186/s12966-020-00987-8 (PMC7336091; doi:10.1186/s12966-020-00987-8)
Supplement: Supplementary file 3 — Additional file 3: Table 1. Inside and outside hobbies during COVID-19 virus outbreak. Table 2. New or returning family hobbies during the COVID-19 virus outbreak. Table 3. Use of online resources during the COVID-19 virus outbreak. [file 12966_2020_987_MOESM3_ESM.docx]

**Additional File 3. Table 1.** Inside and outside hobbies during COVID-19 virus outbreak

| **Activity Option (code)** | ***N* (%)** |
| --- | --- |
| ***Inside hobbies***  (742 respondents; 894 responses provided; †=screen activities, ‡=active activities) | |
| Arts & crafts | 195 (12.9) |
| Puzzles and games | 162 (11.3) |
| Building stuff | 11 (0.8) |
| Cleaning | 4 (0.3) |
| Computer† | 35 (2.4) |
| Cooking/baking | 32 (2.1) |
| Designing/creating | 8 (0.6) |
| Helping others | 1 (0.1) |
| Imaginative play | 9 (0.6) |
| Tablet† | 12 (0.8) |
| Learning | 12 (0.8) |
| Lego | 51 (3.4) |
| Making videos† | 3 (0.2) |
| Music (practice, singing, listening) | 28 (2.0) |
| Physical activity – other (dancing, treadmill, running around, fitness, exercise) ‡ | 37 (2.6) |
| Phone† | 2 (0.2) |
| Reading | 67 (4.5) |
| Sewing | 1 (0.7) |
| Sleeping | 1 (0.1) |
| Television† | 47 (3.2) |
| Toys | 18 (1.2) |
| Video chat† | 7 (0.5) |
| Video games† | 149 (10.2) |
| Writing | 2 (0.1) |
| **Total** | **894 (61**.**8)** |
|  | |
| **Activity Option (code)** | ***N* (%)** |
| ***Outside hobbies***  (334 respondents; 361 responses provided) | |
| Biking | 90 (6.1) |
| Gardening | 11 (0.7) |
| Imaginative play | 4 (0.3) |
| Other (drawing, reading, painting, bird watching) | 27 (1.9) |
| Playing | 12 (0.8) |
| Running | 17 (1.1) |
| Scooting | 11 (0.7) |
| Skating | 12 (0.8) |
| Skipping | 3 (0.2) |
| Snow activities | 2 (0.1) |
| Sport activities | 53 (3.5) |
| Stretching/yoga | 3 (0.2) |
| Trampoline | 21 (1.3) |
| Walking/hiking | 83 (5.5) |
| Yard work | 12 (0.8) |
| **Total** | **361 (24**.**2)** |

**Additional File 3. Table 2.** New or returning family hobbies during the COVID-19 virus outbreak

| **Activity Option (code)** | ***N* (%)** |
| --- | --- |
| ***New or returning family hobbies***  (273 responses) | |
| Arts and crafts | 65 (4.4) |
| Beauty or spa | 2 (0.1) |
| Puzzles and games | 104 (7.1) |
| Building stuff | 1 (0.1) |
| Cleaning or chores | 11 (0.7) |
| Cooking or baking | 1 (0.1) |
| Dancing | 2 (0.1) |
| Educational activities | 3 (0.2) |
| Gardening or landscaping | 3 (0.2) |
| Imaginative play | 2 (0.1) |
| Lego | 4 (0.3) |
| Meals together | 6 (0.4) |
| Movie time | 5 (0.3) |
| Music (practice) | 4 (0.3) |
| Outdoors | 17 (1.2) |
| Physical activities | 14 (1.0) |
| Reading | 8 (0.5) |
| Screens (tablets, computer, television (TV)) | 9 (0.6) |
| Sewing | 1 (0.1) |
| Toys | 3 (0.2) |
| Video games | 7 (0.5) |
| Writing | 1 (0.1) |
| **Total** | **273 (18**.**5)** |

**Additional File 3. Table 3.** Use of online resources during the COVID-19 virus outbreak

| **Activity Option (code)** | ***N* (%)** |
| --- | --- |
| ***Online resources***  (242 responses) | |
| Beachbody app | 5 (0.3) |
| Cosmic Kid Yoga | 1 (0.1) |
| Dance Dance Revolution (DDR) | 1 (0.1) |
| FitBit app | 2 (0.1) |
| GoNoodle app | 13 (0.9) |
| Google Classroom | 9 (0.6) |
| Government website | 2 (0.1) |
| Home workout videos | 9 (0.6) |
| Instagram | 28 (1.9) |
| IXL | 1 (0.1) |
| Joe Wicks | 1 (0.1) |
| Just Dance | 2 (0.1) |
| Khan Academy | 1 (0.1) |
| LesMills (Goodlife) app | 3 (0.2) |
| Mindspace | 1 (0.1) |
| Netflix | 15 (1.0) |
| Nike Training app | 6 (0.4) |
| Online workout | 52 (3.5) |
| ParticipACTION app | 6 (0.4) |
| Peleton | 2 (0.1) |
| PopSugar | 5 (0.3) |
| School-based resources | 18 (1.2) |
| Wii | 5 (0.3) |
| YMCA | 3 (0.2) |
| YouTube | 38 (2.6) |
| Zoom | 14 (1.0) |
| **Total** | **242 (16**.**4)** |
